# Giant Left Ventricular Thrombus Rapidly Developing in an Extremely Short Period of Time in a Patient With Severe Systolic Dysfunction

**DOI:** 10.7759/cureus.61752

**Published:** 2024-06-05

**Authors:** Shun Kawamoto, Naoyuki Otani, Satoshi Mizuguchi, Takashi Tomoe, Takanori Yasu

**Affiliations:** 1 Department of Emergency and General Medicine, Dokkyo Medical University Nikko Medical Center, Nikko, JPN; 2 Department of Cardiology, Dokkyo Medical University Nikko Medical Center, Nikko, JPN; 3 Department of Cardiovascular Medicine and Nephrology, Dokkyo Medical University Nikko Medical Center, Nikko, JPN

**Keywords:** anticoagulants, echocardiography, pulmonary thrombi, acute limb ischemia, left ventricular thrombus

## Abstract

Although left ventricular thrombi (LVTs) are closely related to the prognosis of patients with systolic dysfunction, anticoagulation therapy is not recommended for the primary prevention of LVTs in patients with sinus rhythm heart failure. We report a case of a patient with systolic dysfunction who developed a giant LVT in an extremely short period of time (one month) after an infection. The LVT led to acute limb ischemia, gangrene, and death. Additionally, we incidentally detected pulmonary thrombosis in this patient.

## Introduction

Based on Virchow’s triad of thrombogenesis, the pathogenesis of left ventricular thrombus (LVT) is commonly accepted to involve the interaction of three factors: (i) stasis due to impaired ventricular function, (ii) endocardial damage, and (ii) inflammation/hypercoagulability. In cardiac diseases such as dilated cardiomyopathy (DCM), the contractility of the left ventricle (LV) is weakened, and the LV dilates; accordingly, the blood flow in the LV becomes stagnant, resulting in thrombus formation. Therefore, patients with DCM are prone to systemic embolism complications, a known factor that worsens prognosis [1]. In most patients, the thrombus is thought to develop over a chronic course.

In this report, we describe the case of a patient with reduced LV contractility and a dilated LV, in whom a large, non-mobile thrombus (3 cm in length) formed rapidly in the LV apex over the course of one month and led to acute limb ischemia (ALI), gangrene, and death. A massive thrombus was also found contemporaneously and incidentally at the origins of the bilateral pulmonary arteries. Notably, the LVT had not been detected a month earlier and developed within an extremely short period of time, leading to ALI.

## Case presentation

An 80-year-old woman presented to our hospital with a fever and appetite loss. She had a history of mental retardation and had been a resident of an assisted living facility for people with disabilities for 30 years. Five years prior to presentation, she was diagnosed with a decreased cardiac function and received medical therapy. Notably, she had no history of coronary risk factors, such as hypertension, dyslipidemia, type 2 diabetes, or smoking. Moreover, she had no family history of cardiac diseases, including DCM. 

Five months prior to the most recent presentation, she was hospitalized for congestive heart failure (CHF). Based on echocardiographic findings, we suspected DCM. However, confirmation through cardiac catheterization proved challenging due to her intellectual disability, which hindered her ability to follow resting instructions and maintain a supine position for extended periods (particularly due to acquired degenerative kyphosis). Therefore, ischemic cardiomyopathy could not be ruled out, and the diagnosis of DCM could not be confirmed. She was discharged after approximately three weeks. However, two months prior to the most recent presentation, she was readmitted for a second incident of CHF. She responded well to heart failure treatment, which allowed her to return to independent living with a wheelchair. Pre-discharge echocardiography revealed LV diffuse wall-motion reduction, an LV end-diastolic volume (LVEDV) of 105 mL, an LV end-systolic volume (LVESV) of 73 mL, a low LV ejection fraction (EF) of 30%, moderate mitral regurgitation (MR), and moderate tricuspid regurgitation (TR) without complications of pulmonary hypertension (as evidenced by a TR pressure gradient of 25 mmHg). Detailed observation revealed no LVTs, and anticoagulation therapy was not initiated.

**Figure 1:**
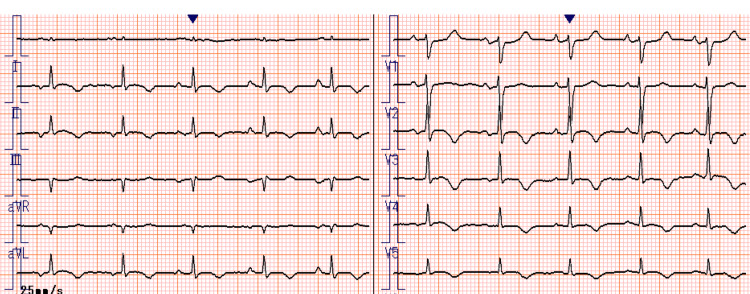
A 12-lead electrocardiogram at admission reveals sinus rhythm and negative T waves in leads II, III, aVF, and V3–V6

Two weeks prior to the most recent presentation, she visited our clinic for fever and loss of appetite, accompanied by facility staff. On admission, her vitals were as follows: body temperature, 37.2℃; blood pressure, 118/53 mmHg; pulse rate, 107 beats/minute; and peripheral oxygen saturation, 91% on room air. Physical examination revealed diminished breath sounds in the right lung field but no rales, murmurs, third heart sounds, edema, or other abnormalities. A 12-lead electrocardiogram (ECG) revealed sinus rhythm with sporadic supraventricular extrasystoles and negative T waves in leads II, III, aVF, and V3-V6 (Figure 1). Compared with the ECG taken at the previous admission, this ECG did not reveal any obvious changes or any findings indicating new cardiovascular events (such as ischemic heart disease). Laboratory testing revealed a white blood cell count of 11,330/μL and an elevated C-reactive protein level of 9.86 mg/dL. Pneumococcal urinary antigen testing was positive for *Streptococcus pneumoniae*. Chest computed tomography (CT) revealed an infiltrating shadow in the upper lobe of the right lung. The patient was diagnosed with pneumonia, and empiric intravenous antibiotics were administered.

**Figure 2:**
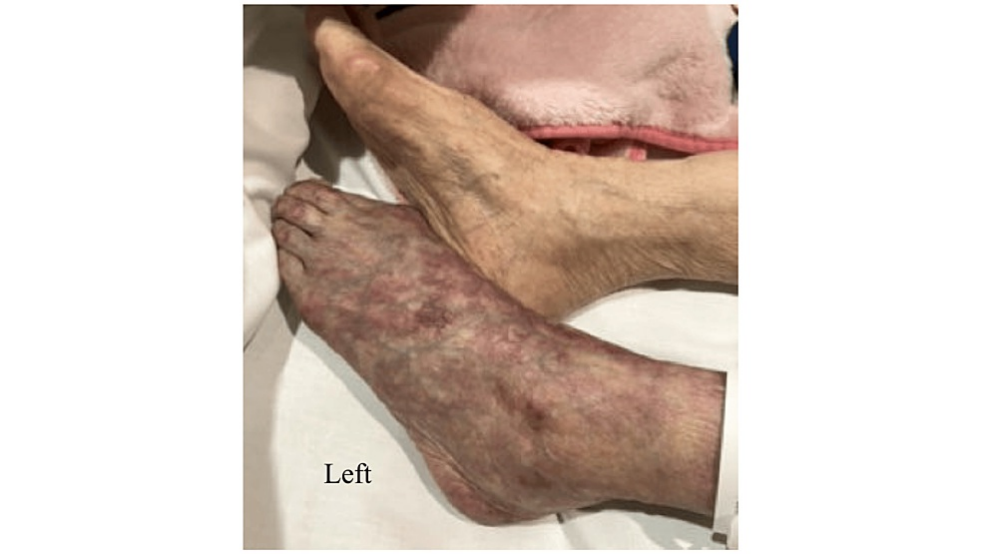
Skin tone changes around the left ankle at onset

**Figure 3:**
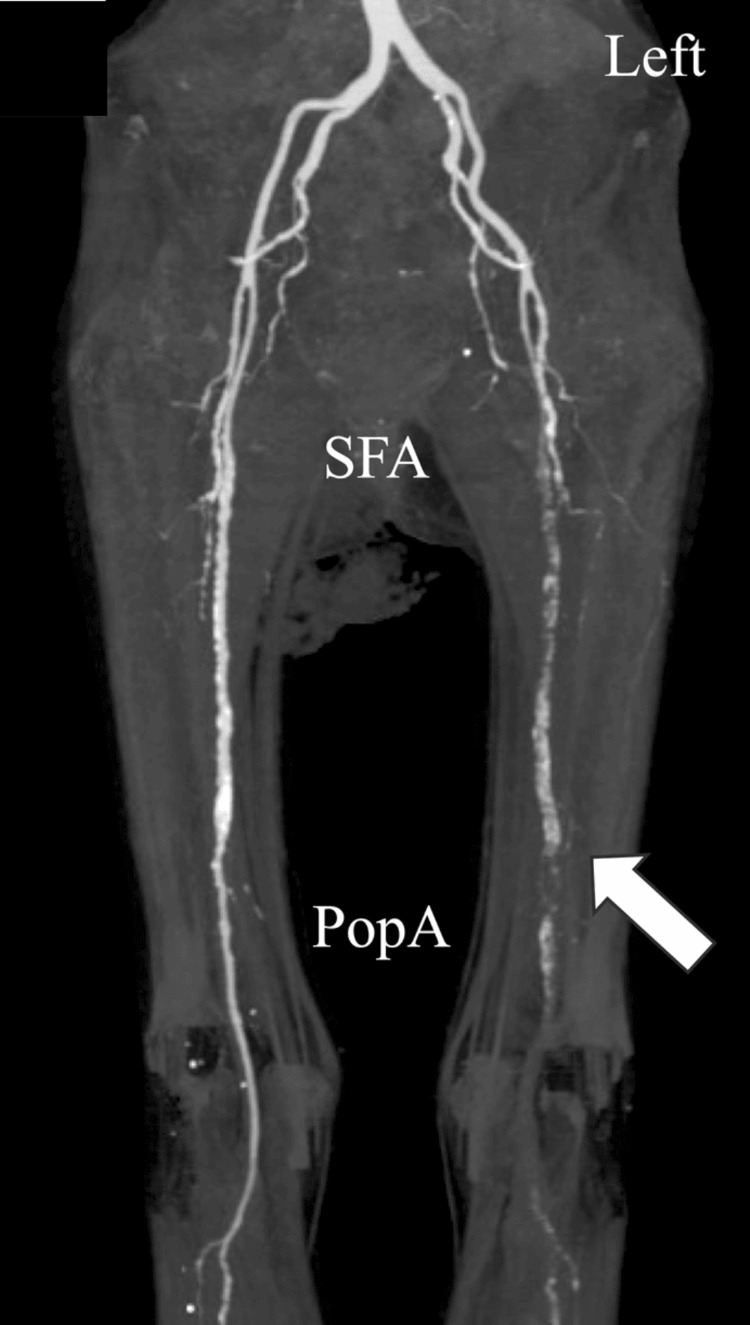
Contrast-enhanced CT performed on day 7 shows obstruction of the left popliteal artery (white arrow) PopA, popliteal artery; SFA, superficial femoral artery

On the sixth day of admission, at midnight, the skin surrounding the left ankle and extending up the lower leg began to show signs of ischemia, exhibiting blackening despite the absence of subjective symptoms (Figure 2). No edema was observed in the bilateral lower legs. We suspected ALI in the left lower limb and performed urgent contrast-enhanced CT the next morning, which revealed a loss of contrast effect peripheral to the left shallow femoral artery, thereby confirming ALI (Figure 3).

**Figure 4:**
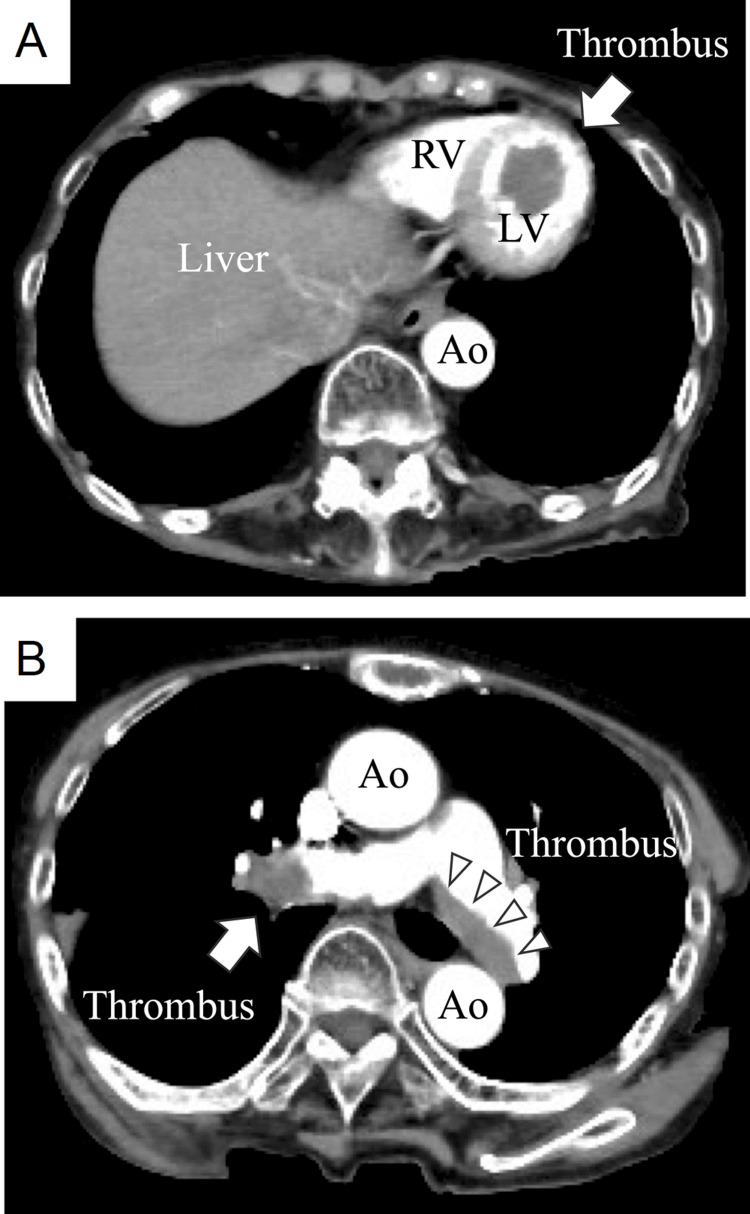
CT shows (A) a giant 30×18 mm thrombus in the left ventricular apex (white arrow) and (B) A new large thrombus in the right pulmonary artery (white arrow) and a mural thrombus in the left pulmonary artery (white arrowheads) Ao, aorta; LV, left ventricle; RV, right ventricle

Further, CT also revealed a massive thrombus within the LV (Figure 4A), raising suspicion that this thrombus was the source of the embolism. The D-dimer level was markedly elevated at 253.6 μg/mL. At admission, the creatine kinase level was 47 U/L; however, it had elevated to 1,358 U/L, with a peak value of 5,083 U/L. Additionally, a large, recently formed thrombus was discovered in the proximal portion of the bilateral pulmonary arteries (Figure 4B). Due to limitations in differentiating new thrombi from the old, it remained unclear whether these were pre-existing or newly formed thrombi. No evidence of deep vein thrombosis (DVT) was observed at this time.

**Figure 5:**
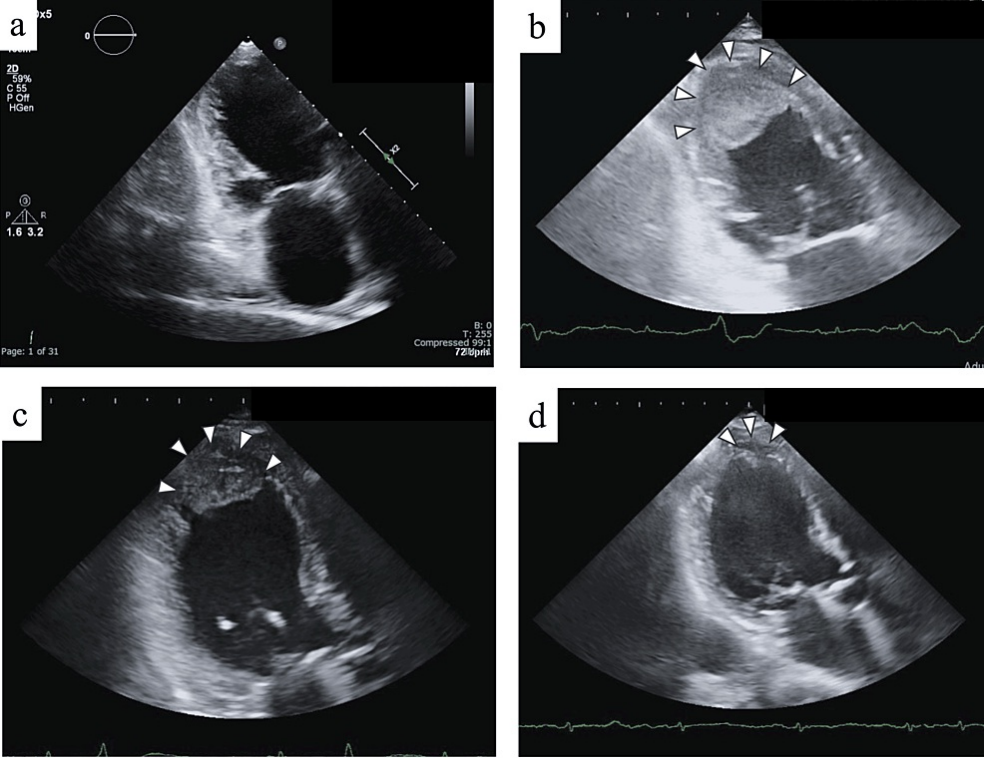
Time course of thrombus images by echocardiography Echocardiographic images approaching from the apex of the heart. This is characterized by a regular shape, uniform echogenicity, and no evidence of mobility, consistent with a fresh thrombus image. (a) Echocardiographic image obtained 40 days before admission. An enlarged left ventricle is seen with a spherical apex without a thrombus. (b) Echocardiographic image obtained seven days after admission (immediately after the onset of the disease). A large thrombus of size 30×18 mm (white arrowheads) is seen in the left ventricular apex. (c) Echocardiographic image obtained on the 14th day after admission (seven days after the onset of the disease). The thrombus appears to have shrunk to 27×13 mm (white arrowheads) with anticoagulation therapy. (d) Echocardiographic image obtained on day 42 after admission (day 35 after the onset of the disease). The thrombus appears to have shrunk to 12×8 mm (white arrowheads).

Transthoracic echocardiography was not performed, as no symptoms of worsening heart failure were noted on admission; however, when CT revealed LVT, echocardiography was urgently performed for confirmation. Although no thrombus was detected 40 days before admission (Figure 5a), repeat echocardiography performed on the seventh day of admission revealed a giant LVT (30×18×24 mm) lodged in the LV apex (Figure 5b). We suspected that this thrombus had led to an embolism in the left popliteal artery. Consistent with previous findings, the LV apex was also spherically enlarged, and the wall motion was diffusely and markedly reduced (LVEDV, 78 mL; LVESV, 58 mL, and EF, 25%), with an enlarged right atrium and ventricle, mild MR, and moderate TR (TR pressure gradient, 25 mmHg). Within the scope of investigation in transthoracic echocardiography, no atrial or ventricular septal defects were observed. 

Upon diagnosis of ALI, we initiated heparin treatment as soon as possible [2]. The patient did not complain of pain and showed no evidence of muscle weakness. Therefore, we could not examine her accurately using the Rutherford classification for ALI [3]. The patient and her family were informed about her condition and the treatment options, including thromboembolization by a balloon catheter, revascularization by bypass surgery or endovascular treatment, and lower limb amputation. The patient and her family did not consent to any surgical intervention. Given that tissue plasminogen activator is not available for ALI in Japan, treatment with heparin was continued, followed by the addition of warfarin the next day, aiming for a prothrombin time-international normalized ratio of 2.0-3.0. Anticoagulation therapy was administered, which resulted in a reduction in the LVT size to 27×13 mm at 14 days and 12×8 mm at 42 days after admission (Figures 5b-5d). However, gangrene with ischemia developed in the left lower extremity, subsequently leading to an infection. Therapy with warfarin, fluids, and antibiotics was continued; however, the patient died of sepsis 54 days after admission. 

## Discussion

We described the course of a patient with systolic dysfunction who developed a rapidly progressing giant thrombus in the LV that was triggered by an infection. Although LVTs are well-known complications in patients with systolic dysfunction, cases of rapid thrombus formation over the course of a month are rare. Notably, the patient’s readmission shortly after discharge facilitated close monitoring of thrombus progression, which offered valuable insights. To date, no reports are available on how long a thrombus can take to develop in the LV.

Gottdiener et al. reported that in patients with chronic DCM, the incidence of LVT on echocardiograms was 36% (44/123 patients), and that of systemic embolic events was 11% (11/96 patients) [4]. Despite advances in anticoagulation therapy, no study has found a significant decrease in the LVT risk in patients receiving it [1]. In one study, LVT was less common in patients with non-ischemic cardiomyopathy (NICM), including DCM, than in those with ischemic cardiomyopathy [5]. The EFs were lower and LVs were larger in patients with NICM with LVT than in those with ischemic cardiomyopathy with LVT and those with NICM without LVT. Notably, many patients with NICM with LVT also developed thrombi in the cardiac chambers other than the LV, at a frequency 10-fold higher than that in patients with ischemic cardiomyopathy with LVT [5]. These patients also exhibited left ventricular tachycardia and concurrent pulmonary thrombosis. This suggests the need to distinguish between ischemic cardiomyopathy and NICM. However, unfortunately, we could not rule out ischemic cardiomyopathy.

Patients with heart failure often have multiple risk factors that amplify the risk of DVT [6,7]. Systolic dysfunction and LV dilation lead to blood stasis, triggering coagulation system activation and fibrin production, ultimately increasing the DVT risk. In our patient, DVT detection was inadequate due to gangrene in the lower extremities; however, we speculated that DVT emerged from pneumonia, and the resulting decline in activities of daily living led to pulmonary thrombosis. The patient did not exhibit abnormalities in the protein S and C antigen levels and activity or in lupus anticoagulants, which indicated an inherited thrombophilic predisposition or elevated antiphospholipid antibodies; this suggested an acquired thrombophilic predisposition. The antithrombin III level was 47% (normal range, 79-121%).

Cardiogenic embolisms, such as stroke and peripheral arterial embolisms, are closely associated with heart failure. However, the 2012 American College of Chest Physicians guidelines on antithrombotic therapy and prevention of thrombosis also recommend the use of antiplatelet therapy or warfarin in patients with systolic dysfunction without established coronary artery disease and LVT (grade 2C) [8]. Thus, the detection of LVT is very important and requires periodic echocardiographic examinations. However, to date, no clear evidence regarding the frequency of these checkups has been reported. In patients with acute anterior myocardial infarction with systolic dysfunction, the follow-up time for echocardiography is seven days after the onset of the infarction or within a few weeks after the infarction if discharged earlier [9]. However, this has only been reported in the acute phase of thrombus-prone ischemic heart disease, and evidence indicating that repeated echocardiography leads to a reduction in embolic events is insufficient. 

We believe that in our patient, inflammation, in addition to stasis caused by contractile dysfunction, contributed to thrombus formation. Acute systemic respiratory tract infections, including viral and bacterial infections, are associated with a transient increase in the risk of vascular events [10]. Particularly, patients with COVID-19 have an increased incidence of thromboembolic complications. Similar to our case, a case of simultaneous pulmonary, intracardiac, and peripheral arterial thrombosis in a patient with COVID-19 has been reported [11]. In addition, a case of systolic dysfunction with thrombi in both ventricles has also been reported in a patient with COVID-19 [12]. Furthermore, bacteremia and severe pneumonia are associated with an increased incidence of vascular events, including myocardial infarction and stroke [13,14]. This suggests that it is not the infection itself, but the subsequent host immune reaction that is the predominant trigger of macrovascular thrombotic events [15]. Therefore, we suggest that if a patient with systolic dysfunction becomes hypercoagulable owing to inflammation or other factors, then the presence of a thrombus or pulmonary hypertension should be promptly determined using echocardiography.

A limitation of our case report is that we could not confirm the diagnosis of DCM, as ischemic heart disease was not ruled out. Ischemic heart disease increases the risk of thromboembolism, indicating the importance of the diagnosis of ischemic heart disease when managing patients with severe systolic dysfunction. As this is a case report, further studies with large sample sizes are warranted to validate our findings. In particular, future studies with more cases of LVT should be conducted to examine the causal relationship between inflammation and thrombus formation.

## Conclusions

In the present case, a patient with systolic dysfunction without LVT developed a giant thrombus within a short period of time (i.e., one month), triggered by an infection. If a patient with systolic dysfunction develops a hypercoagulable state, including an inflammatory disease, checks should be done for LVT and other thrombi immediately because thrombosis can develop extremely rapidly. Prevention of LVT is important for improving life outcomes in patients with systolic dysfunction; however, anticoagulation therapy is not recommended for the primary prevention of LVT. 
